# Rlip Depletion Alters Oncogene Transcription at Multiple Distinct Regulatory Levels

**DOI:** 10.3390/cancers14030527

**Published:** 2022-01-21

**Authors:** Ashly Hindle, Chhanda Bose, Jihyun Lee, Philip T. Palade, Christopher J. Peterson, P. Hemachandra Reddy, Sanjay Awasthi, Sharda P. Singh

**Affiliations:** 1 Department of Internal Medicine, Texas Tech University Health Sciences Center, Lubbock, TX 79430, USA; ashly.hindle@ttuhsc.edu (A.H.); chhanda.bose@ttuhsc.edu (C.B.); jihyun.lee@ttuhsc.edu (J.L.); christopher.peterson@ttuhsc.edu (C.J.P.); hemachandra.reddy@ttuhsc.edu (P.H.R.); 2 Division of Hematology & Oncology, Department of Internal Medicine, Texas Tech University Health Sciences Center, Lubbock, TX 79430, USA; 3 Department of Pharmacology and Toxicology, University of Arkansas for Medical Sciences, Little Rock, AR 72205, USA; ppalade@uams.edu; 4 Department of Pharmacology and Neuroscience, Texas Tech University Health Sciences Center, Lubbock, TX 79430, USA; 5 Department of Public Health, Graduate School of Biomedical Sciences, Texas Tech University Health Sciences Center, Lubbock, TX 79430, USA; 6 Department of Neurology, Texas Tech University Health Sciences Center, Lubbock, TX 79430, USA; 7 Department of Speech, Language and Hearing Sciences, Texas Tech University Health Sciences Center, Lubbock, TX 79430, USA; 8 UMC Cancer Center, UMC Health System, Lubbock, TX 79415, USA

**Keywords:** RALBP1, Rlip, methylation, transcription, regulation, breast cancer, lung cancer, CpG island

## Abstract

**Simple Summary:**

Rlip76 is a multifunctional membrane protein that facilitates cancer growth, and its depletion kills cancer cells. We recently found that Rlip depletion also results in broad changes to oncogene and tumor suppressor transcription. The present studies were designed to decipher the unknown downstream signaling pathways and transcriptional regulatory mechanisms driving the effect. Building on prior findings that Rlip depletion induces broad methylomic changes, we found using bioluminescence reporter assays that depletion of Rlip also exerts transcriptional control over several cancer genes through methylation-independent changes in transcription factor-mediated activation of their promoter regions and through additional as yet unidentified mechanisms. These findings have important implications for Rlip-targeted cancer therapy.

**Abstract:**

Rlip76 (Rlip) is a multifunctional membrane protein that facilitates the high metabolic rates of cancer cells through the efflux of toxic metabolites and other functions. Rlip inhibition or depletion results in broad-spectrum anti-cancer effects in vitro and in vivo. Rlip depletion effectively suppresses malignancy and causes global reversion of characteristic CpG island methylomic and transcriptomic aberrations in the p53-null mouse model of spontaneous carcinogenesis through incompletely defined signaling and transcriptomic mechanisms. The methylome and transcriptome are normally regulated by the concerted actions of several mechanisms that include chromatin remodeling, promoter methylation, transcription factor interactions, and miRNAs. The present studies investigated the interaction of Rlip depletion or inhibition with the promoter methylation and transcription of selected cancer-related genes identified as being affected by Rlip depletion in our previous studies. We constructed novel promoter CpG island/luciferase reporter plasmids that respond only to CpG methylation and transcription factors. We found that Rlip depletion regulated expression by a transcription factor-based mechanism that functioned independently of promoter CpG methylation, lipid peroxidation, and p53 status.

## 1. Introduction

Rlip76 (Rlip, the 76 kDa isoform encoded by the *RALBP1* gene at human genomic locus 18p11.22) has been well-established by several lines of evidence as a permissivity factor required for oncogenic transformation, cancer growth, and invasion/metastasis [1,2,3,4,5,6,7]. Rlip contributes to, or is necessary for, many aspects of cell physiology, including clathrin-dependent endocytosis (CDE), receptor tyrosine kinase signaling, the efflux of GSH conjugates of toxic oxidative metabolite 4-hydroxynonenal (4-HNE) and xenobiotics, and mitochondrial fission [8,9,10]. Rlip often participates in functions that necessitate mechanical forces such as constriction or cytoskeletal motion, as might be expected for an effector of the ras-related proteins RALA and RALB [11]. Among patients of the METABRIC breast cancer study, Rlip mutations were not observed and homozygous deletion of *RALBP1* was exceedingly rare (0.09% of patients), supporting the importance of normally functioning Rlip protein in oncogenic processes [12]. Rlip depletion or inhibition inhibits the growth of breast, lung, colon, kidney, and prostate cancer as well as neuroblastoma in xenografts using immune-deficient mouse models and in syngeneic melanoma implants in immune-sufficient mice [12,13,14,15,16,17,18,19]. It is not currently known which of the many aspects of Rlip physiology are causal with respect to the remarkable broad-spectrum anticancer activity seen with Rlip depletion or inhibition. Recently, it has become clear that Rlip inhibition also results in global methylomic and transcriptomic changes, affecting many pathways, including several cancer-related pathways [1]. These methylomic changes included both methylation and demethylation events at both promoter CpG islands and gene body CpG dinucleotides.

A genomic CpG dinucleotide is a cytosine followed by a guanine in the 5′→3′ direction and is distinct from a 5′→3′ GpC dinucleotide. CpG islands were classically defined by Gardiner-Garden and Frommer as a stretch of at least 200 base pairs with a GC content greater than 50% and an observed/expected CpG ratio greater than 0.60. Since then, other criteria for CpG islands have also been explored, and the resulting estimations place the fraction of gene promoters associated with CpG islands at ~50% to ~70% in humans [20,21,22,23]. CpG islands are typically located in the promoter regions of genes and commonly span from upstream of the coding sequence, through the first exon, and into the first intron. However, CpG islands can also occur in gene bodies downstream of promoters or in intergenic regions [20]. Methylation of cytosine C5 residues in promoter CpG islands typically results in stable gene repression; although, this is not universally the case, particularly with cancer, where aberrant hypermethylation can result in overexpression [24,25]. While promoter methylation changes are typically considered to be a cause of expression changes, there is also evidence that transcriptional activation by transcription factors can precede methylation changes, suggesting that promoter methylation changes can be both a cause and an effect of transcriptional changes [26,27]. Silencing of tumor suppressors and DNA repair enzymes by aberrant hypermethylation is seen in many cancers, and the DNMT1 inhibitors azacitidine and decitabine are used to exploit this fact in hematologic cancers to demethylate suppressed promoters, thereby reactivating silenced tumor suppressors [28]. In contrast to the methylation of promoter CpGs, which is typically characterized as suppressing in nature, methylation of gene body CpGs is typically characterized as activating in nature. Interestingly, while DNMT1 inhibitors can lift the repression of tumor suppressors by promoter demethylation, they can also repress oncogenes that have been activated by gene body methylation, thereby normalizing the expression of both tumor suppressors and oncogenes [28,29]. As DNMT1 functions during DNA replication to copy methylation patterns from parental strands to daughter strands, DNMT1 inhibitors are effective against rapidly cycling cells of hematological malignancy, but have limited effectiveness against solid tumors, which generally replicate more slowly [28].

Rlip inhibition or depletion, by contrast, has considerable activity against xenografted solid tumors in addition to considerable overlap with the methylomic and transcriptomic effects induced by DNMT1 inhibitors. However, Rlip inhibition has the advantage of having no known requirement for DNA replication to induce cytotoxicity. Rlip inhibition may induce its anticancer effects against solid tumors through normalization of tumor suppressor and oncogene expression, disruption of other important Rlip-mediated functions that are critical to cancer cells, or combinations of these effects. Despite the compelling case for the further development of Rlip inhibitors, owing to the impressive broad-spectrum antitumor effects and the pleiotropic functions of Rlip in cancer cells, additional information is needed to understand the mechanisms underlying the striking transcriptomic and epigenetic changes. Cells have several layers of transcriptomic regulation at their disposal including histone modifications, CpG methylation, transcription factors, microRNAs, and long non-coding RNAs. Small changes at the level of a single protein that participates in multiple protein–protein complexes can cause profound changes in the relative ratios of these complexes and disproportional phenotypic effects, referred to as haploinsufficiency phenomena, as is seen with p53-binding proteins such as MDM2 and MDM4 [30]. Rlip has been shown to interact with p53, and the near complete suppression of spontaneous malignancy in p53 null (p53^−/−^) mice by only heterozygous deficiency of Rlip led us to propose that a similar haploinsufficiency mechanism may exist involving Rlip, p53, and another common binding partner of both, perhaps HSF1 [1]. This is strongly supported by recent studies showing that heterozygous Rlip deficiency suppresses Her2-driven murine breast cancer (the MMTV-ERBB2 mouse model) as well [31]. The specific protein complexes and their molecular functions remain to be elucidated, but breast tumors from MMTV-ERBB2 mice frequently have mutations in p53, a phenomenon that is also prevalent in human HER2-positive breast cancers [32,33]. In summary, Rlip depletion causes broad transcriptomic and methylomic changes, which could result from a number of effects including altered levels or functions of transcription factors, DNA methylation enzymes, or other regulatory/signaling protein complexes, as well as a generalized shift in the intracellular physiological milieu due to the sustained oxidative or genotoxic stresses of 4-HNE accumulation.

In this work, we attempted to elucidate the roles of these possible mechanisms through which Rlip depletion or inhibition may interact with CpG island methylation to regulate the expression of selected cancer-related genes identified in previous studies as being affected by Rlip deficiency. The genes were selected on the basis of established interactions with known Rlip functions in promoting cancer cell survival or growth. Fibroblast growth factor 8 (FGF8) is a growth factor that activates the FGFR receptors and has roles in breast, prostate, and ovarian cancers [34,35,36]. We have previously shown that Rlip knockdown interrupts FGF signaling, resulting in the loss of STAT3 nuclear translocation, while Rlip overexpression increased nuclear STAT3 [1]. Mitogen-activated protein kinase 14 (MAPK14, aka p38) contributes to aggressive disease in hepatocellular carcinoma, gastric cancer, and renal cell carcinoma [37,38,39]. MAPK14 is activated by FGF/FGFR signaling and has been shown to, in turn, activate FGFR and EGFR internalization [40]. Protein kinase C alpha (PRKCA) is associated with metastasis and poor outcome in breast cancer and lung cancer patients [41,42]. PRKCA regulates the transport activity of Rlip in lung cancer, and Rlip is an important effector of activities of PRKCA, which promote the survival, growth, and drug-resistance of cancer cells in vitro as well as phorbol ester-promoted carcinogenesis in vivo [43,44]. Peroxisome proliferator activated receptor alpha (PPARA) functions in lipid metabolism and is of interest because Rlip depletion affects metabolic syndrome, obesity, and insulin resistance [45,46,47]. PPARA is a lipid-responsive transcription factor that induces the production of peroxisomes and the regulation of fatty acid metabolism. Polymorphisms in PPARA have been associated with breast cancer risk [48]. PPARA is constitutively upregulated in Rlip knockout mice [45]. CREB-binding protein (CREBBP) is a coactivator of many transcription factors, including NRF2, which plays a role in the cellular response to oxidative stress [49,50]. Rlip expression is driven by P300, a related CREB-binding coactivator with considerable homology and functional overlap with CREBBP [51,52]. Both CREBBP and P300 possess histone acetyltransferase activity, which facilitates transcription by decreasing DNA compaction [53]. Leucine-rich repeat protein 1 (LRR1) suppresses activation of NF-Kappa B. High LRR1 expression increases the mortality of liver cancer and renal cancer [54]. Rlip knockdown interferes with the regulation of several plasma membrane receptors that rely on CDE, including TNF receptors, thus functional overlap between Rlip and LRR1 is probable [55,56]. Protein kinase C zeta (PRKCZ) is an atypical PKC that is activated by phosphatidylserine, but is insensitive to diacylglycerol or calcium. PRKCZ mediates motility in pancreatic and ovarian cancer cells [57,58].

In this work, we use mRNA expression and luciferase expression by novel CpG-free promoter/luciferase reporter plasmids to address several hypotheses regarding the nature of the methylomic and transcriptomic regulation previously observed following Rlip knockdown. By studying the mRNA expression of these genes, we observed the summated effects of all operative regulatory inputs that affect the transcriptome, and the promoter/luciferase reporter plasmid constructs allowed us to focus in on only methylation-based and transcription factor-based regulation, as such plasmids are insensitive to regulation by histone modifications; microRNAs; long non-coding RNAs; or other factors affecting transcript-specific mRNA splicing, base editing, or stability. Using these tools, we confirmed prior findings that Rlip depletion exerts regulatory transcriptomic influences on cancer-related genes. Our reporter results suggest that, at the 24 h time-point, this regulatory influence operates through a transcription factor-based mechanism rather than through promoter methylation, and this does not appear to depend on p53 status. These luciferase reporter results, in combination with the qRT-PCR results, also point to an additional as yet unidentified layer or layers of regulatory influence that cannot be explained by transcription factors. Additionally, our results suggest that the observed Rlip-responsiveness is not simply due to the oxidative stress of 4HNE accumulation, and is not an artifact of imbalanced cytotoxicity or clathrin-dependent endocytosis resulting from Rlip inhibition. Further studies of the transcriptomic regulatory effects of Rlip depletion are warranted, and studies on transcription factor interactions, histone remodeling, and microRNAs should be prioritized.

## 2. Materials and Methods

### 2.1. Tissue Culture Methods

MCF7 and MDA-MB-231 breast cancer cells were cultured in DMEM supplemented with 10% FBS and 1% Pen-Strep (Gibco). NCI-H358 (H358) lung cancer and NCI-H520 (H520) lung cancer cells were cultured in RPMI supplemented with 10% FBS and 1% Pen-Strep. Puck’s Saline A + EDTA was used to detach adherent cells and was kindly provided by the TTUHSC School of Medicine Cancer Center [59]. Cells were cultured in a 37 °C humidified incubator in 20% O_2_ and 5% CO_2_. MCF7 and MDA-MB-231 are representative of estrogen receptor positive (ER+) and triple negative breast cancer (TNBC), respectively. NCI-H358 is a p53-null non-small cell lung cancer (NSCLC) cell line with a Kirsten rat sarcoma viral proto-oncogene (*KRAS*) mutation. NCI-H520 is a NSCLC cell line with low expression of wild-type p53, which is inducible [60]. These cell lines were chosen because they represent distinct important subsets of patients in their respective cancers, and they have previously been reported to be responsive to Rlip depletion in tumor growth models [12,16]. This diversity of models ensures broad applicability of our findings. All cell lines used in this study are of human origin and were obtained from ATCC (American Type Culture Collection, Manassas, VA, USA).

### 2.2. Target Selection

We have previously reported global methylomic and transcriptomic changes in the livers of p53 knockout mice following Rlip depletion by R508, a phosphorothioated antisense DNA oligonucleotide targeted to the *RALBP1* mRNA [1]. From these datasets, *LRR1* and *PPARA* were selected for further study because of the methylomic and transcriptomic changes following Rlip knockdown by R508 and because they share a functional commonality with Rlip. Additionally, we used reduced representation bisulfite sequencing (RRBS) to examine methylomic changes in NCI-H358 and NCI-H520 lung cancer cell lines following treatment with a proprietary small molecule inhibitor of Rlip. The methylation changes were analyzed using Qiagen’s Ingenuity Pathway Analysis (IPA), and we selected *PRKCA*, *PRKCZ*, *MAPK14*, *CREBBP*, and *FGF8* because they represent critical nodes in the pathways most affected by methylation changes in NCI-H358 or NCI-H520 following Rlip inhibition and because they have previously been reported to have relevance in cancer [34,35,36,39,41,57,61].

### 2.3. Rlip Knockdown and qRT-PCR

Cells were seeded at 70–90% confluency in six-well plates in complete medium. After overnight incubation, plates were transfected with 2.5 µg *RALBP1* antisense locked nucleic acid (Rlip-LNA) Exiqon GapmeRs (Qiagen, Hilden, Germany), or scrambled control, as described previously [12]. Transfection was performed using Lipofectamine 3000 and p3000, diluted in Opti-MEM (Thermo Fisher Scientific, Waltham, MA, USA) according to the manufacturer’s protocol. Vehicle effects on mRNA expression were examined in untreated MCF7 cells or in cells exposed only to Lipofectamine+P3000. Twenty-four hours after treatment, cells were pelleted in ice-cold PBS, and RNA was extracted using the Qiagen RNeasy Mini Kit with quantification by NanoDrop One Spectrophotometer (Thermo Fisher). cDNA was synthesized using the Superscript IV VILO Master Mix (Thermo Fisher) with gDNA digestion according to the manufacturer’s protocol. Primers were selected from the Harvard PrimerBank or designed using NCBI Primer-BLAST, and purchased from Integrated DNA Technologies (Coralville, IA, USA). qRT-PCR primer sequences are shown in Table S1. All primers were verified to amplify only a single product by gel analysis. qRT-PCR plates were analyzed using PowerUp SYBR Green Master Mix (Thermo Fisher). Plates were analyzed using an Applied Biosystems 7900HT Fast Real-Time PCR System (Thermo Fisher) or an Applied Biosystems QuantStudio 12K Flex, and dissociation curves were included for all qRT-PCR experiments. Ct values were determined using the default analysis settings in the Applied Biosystems SDS 2.4 software or in the QuantStudio 12K Flex software v1.4, and normalization was done using the ddCt method.

### 2.4. Rlip Overexpression, Rlip Protein Treatment, and Arachidonic Acid Treatment

We explored the behavior of Rlip-responsive genes after treatments to increase Rlip by protein- or plasmid-based methods and after treatment to increase oxidative stress by exposure to arachidonic acid (AA), an omega-6 polyunsaturated fatty acid precursor of 4HNE. Cells were cultured and seeded overnight in six-well plates as described above for Rlip knockdown. For Rlip overexpression, cells were transfected with 2.5 µg of a pcDNA3.1-based plasmid construct expressing *RALBP1* mRNA under control of a CMV promoter [47,62] or with the empty vector using Lipofectamine 3000 and P3000 according to the manufacturer’s protocol. Cells were collected after 24 h. For Rlip protein treatment, cells were seeded overnight in a six-well plate and treated with 50 µg of recombinant Rlip protein in proteoliposomes [63] (Terapio Corporation, Austin, TX, USA) suspended in PBS or with PBS alone. Cells were collected after 24 h. The treatment exploits Rlip’s capability of functionally integrating into cells when delivered by proteoliposome [50]. For AA treatment, cells were dosed at 150 µM in DMSO or with DMSO alone for 24 h before pellets were collected for qRT-PCR analysis as described above.

### 2.5. Western Blot

Western blot was performed to verify Rlip overexpression using the pcDNA3.1 construct. Cells were seeded and transfected as described above. Protein was extracted using RIPA buffer (Cat# R0278, MilliporeSigma, St. Louis, MO, USA) with cOmplete™ Mini protease inhibitor cocktail (MilliporeSigma, Cat# 11836153001), prepared in Laemmli buffer, and separated by SDS-PAGE using Bolt 4–12% Bis-Tris gels (Thermo Fisher). Protein was transferred to 0.22 µm nitrocellulose (Cat# 1620097, Bio-Rad Laboratories, Hercules, CA, USA) using wet transfer for 1.5 h at 30 V. Blots were probed using commercial antibodies in Pierce Clear Milk Blocking Buffer (Thermo Fisher) in 0.1% TBST and imaged on a VersaDoc imaging system (Bio-Rad). Antibodies were diluted 1:1000 and included the following: anti-RALBP1 (Cat# TA500964, OriGene, Rockville, MD, USA), anti-beta actin (Cat# PA5-16914, Thermo Fisher), HRP-linked mouse IgGκ Binding Protein (Cat# sc-516102, Santa Cruz Biotechnology, Dallas, TX, USA), and anti-rabbit IgG HRP-linked (Cat# 7074S, Cell Signaling Technologies, Danvers, MA, USA).

### 2.6. Construction of pCpGL Firefly Luciferase Reporters

CpG islands from the promoter regions immediately upstream of the translation start sites of the *FGF8*, *PRKCA*, *CREBBP, MAPK14, PRKCZ*, *LRR1*, and *PPARA* genes were cloned from human DNA and inserted into the pCpGL CpG-free firefly luciferase reporter plasmid graciously provided by Dr. Michael Rehli [64]. CpG islands were identified using the UCSC Genome Browser hosted by the Genomics Institute at the University of California, Santa Cruz. Primers were generated using NCBI Primer-BLAST or manual selection, and 5′ restriction sites were chosen so as to have minimal homology to the genomic sequence. All segments were successfully amplified from human DNA by varying the DMSO concentrations in the amplification reactions between 2.5% and 7.5% to reduce the melting temperatures of the GC-rich amplicons. Primer sequences with restriction sites and amplicon lengths are indicated in Table S2. Amplicons were gel purified, ligated into Promega’s pGEM-T plasmid, and transformed by heat shock into competent JM109 *E. coli* (Promega Corporation, Madison, WI, USA) for selection. After restriction digestion from pGEM-T, gel purified inserts were ligated into pCpGL-basic firefly luciferase reporter plasmid and transformed into PIR1 *E. coli* under Zeocin (Thermo Fisher) selection to obtain the final experimental CpG island-luciferase reporter plasmid constructs. The CpG island inserts in the final pCpGL reporter plasmid constructs were validated by restriction digestion (Figure S1) and by sequencing (Genewiz, Chelmsford, MA, USA). M.SssI CpG methyltransferase (New England Biolabs, Ipswich, MA, USA) was used to generate in vitro methylated reporter constructs, along with unmethylated controls. Following reporter plasmid methylation, gel analysis showed complete protection from digestion by HpaII, a methylation-sensitive restriction enzyme, while unmethylated controls showed complete digestion (Figure S2).

### 2.7. Dual Luciferase CpG Island Reporter Assays

Cells were seeded overnight in 65 µL complete medium in white flat bottom 96-well tissue culture plates (PerkinElmer, Waltham, MA, USA) at 15,000 to 35,000 cells per well to attain 70–90% confluency at the time of transfection. Firefly luciferase reporter plasmids and Rlip-LNA or scrambled control were transfected in 10 µL each of Opti-MEM reduced serum medium (Thermo Fisher) using Lipofectamine 3000 with p3000. The pRL-SV40 Renilla luciferase reporter plasmid (Promega) served as an internal standard and was transfected simultaneously with the firefly luciferase reporter plasmids. Owing to the considerable difference in signal intensity between the various reporter constructs and between unmethylated reporters and their methylated counterparts, all wells transfected with different constructs or different treatments were separated by one blank well to reduce the effects of light bleed on wells of lower signal intensity. Firefly and Renilla luciferase signals were analyzed on a SpectraMax ID3 microplate reader (Molecular Devices, San Jose, CA, USA) using the Dual-Glo Luciferase Assay System (Promega) according to the manufacturer’s instructions. The responsiveness to RLIP knockdown by locked nucleic acid (Rlip-LNA, 50–100 ng/well) relative to scrambled control was evaluated for both unmethylated (UM) and in vitro methylated (M) variants of each reporter at 24 h.

### 2.8. MTT Cytotoxicity Assay

MTT assay was performed in parallel to dual luciferase assays using the same cell seeding numbers and volumes, transfection conditions, and plate analysis timepoints as described for the dual luciferase methods. Briefly, additional complete medium was added to a final volume of 200 µL per well, and 40 µL of 5 mg/mL ChemCruz Thiazolyl Blue MTT reagent (Cat# sc-359848A, Santa Cruz) in PBS was added to each well. After 2–3 h incubation at 37 °C, wells were aspirated and 50 µL DMSO was added to each well. Plates were rocked at room temp for 10 min to dissolve MTT crystals before reading on a SpectraMax Plus microplate reader (Molecular Devices).

### 2.9. Statistical Methods and Software

Comparisons of mean normalized mRNA and luciferase expression were done using Student’s *t*-test with significance at *p* < 0.05 using Excel (Microsoft Corporation, Redmond, WA, USA). Correlation analyses used Pearson’s r, performed in GraphPad Prism version 5.02 (GraphPad Software Inc., San Diego, CA, USA). Graphs were created using GraphPad Prism version 5.02. Predicted transcription factor binding sites were identified using PROMO hosted by the ALGGEN server [65,66]. PROMO search criteria were set to identify only sequences that match experimentally verified binding sequences for each transcription factor.

## 3. Results

### 3.1. Rlip Knockdown Regulates Oncogene mRNA Expression

**Figure 1:**
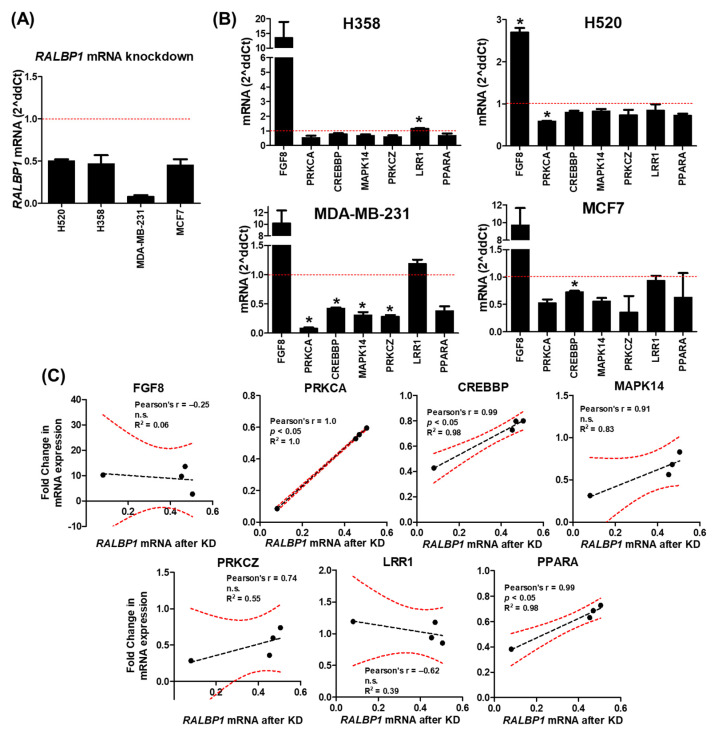
Rlip knockdown regulates oncogene transcription. H358 and H520 lung cancer cells and MDA-MB-231 and MCF7 breast cancer cells were collected 24 h following transfection with a *RALBP1*-targeted locked nucleic acid (Rlip-LNA), which induces RNAse H-mediated mRNA degradation. (**A**) *RALBP1* mRNA knockdown at 24 h following Rlip-LNA transfection. (**B**) Target mRNA expression following Rlip knockdown was evaluated relative to scrambled controls, which were defined as 1 and are indicated by the red dotted lines. (**C**) For *PRKCA*, *CREBBP*, and *PPARA*, the degree of expression change significantly correlated with the observed level of *RALBP1* mRNA knockdown (plotted as the fraction of *RALBP1* mRNA remaining after knockdown (KD)), supporting a causal relationship between Rlip knockdown and transcriptional regulation. In (**B**), asterisks (*) indicate *p* < 0.05 by Student’s *t*-test when comparing mRNA expression from each Rlip-LNA treatment to its corresponding scrambled control treatment. Error bars indicate SEM (*n* = 3).

We selected several Rlip- and cancer-related genes as described in Section 2 in order to study the effects of Rlip depletion on oncogene regulation. Cells were collected 24 h following transfection with a *RALBP1*-targeted locked nucleic acid (Rlip-LNA), which induces RNAse H-mediated mRNA degradation. Rlip-LNA induced 50–92% knockdown in *RALBP1* mRNA at 24 h following transfection (Figure 1A). FGF8 mRNA expression showed apparent increases in all cell lines at 24 h following Rlip-LNA transfection (Figure 1B), although the change was only statistically significant (*p* < 0.05) for H520. *PRKCA*, *CREBBP*, *MAPK14*, *PRKCZ*, and *PPARA* mRNA showed apparent decreases across the four cell lines following Rlip knockdown, although the changes for a given gene were statistically significant (*p* < 0.05) in only certain cell lines, as indicated by the asterisks. *LRR1* showed only minor changes and no consistent response across the four cell lines. The degree of transcriptional regulation exerted by Rlip knockdown significantly correlated (*p* < 0.05) with the degree of Rlip depletion for *PRKCA*, *CREBBP*, and *PPARA* (Figure 1C). *MAPK14* and *PRKCZ* showed non-significant positive relationships between the degree of Rlip depletion and degree of expression change. These results support a causal relationship between Rlip depletion and transcriptional regulation. FGF8 was substantially upregulated in all cell lines irrespective of the degree of Rlip depletion. As an additional quality control, we wanted to observe any effects that the Lipofectamine 3000 and P3000 transfection reagents might have on expression. To study this, we evaluated the expression in completely untreated control cells and in cells exposed to Lipofectamine 3000 and P3000 (L3K+P3000) only. Figure S3 shows mRNA expression in untreated MCF7 cells and in cells exposed to L3K+P3000 vehicle only, alongside that of cells transfected with Rlip-LNA or scrambled control, all normalized to the untreated cells (defined as 1) for equivalent comparison. For *FGF8*, *PRKCA*, *CREBBP*, and *LRR1*, no change in mRNA expression was observed following exposure to L3K+P3000. Moderate deceases in mRNA were observed for *MAPK14*, *PRKCZ*, and *PPARA* in response to L3K+P3000. However, the direction of the regulatory effects on target expression following Rlip knockdown by LNA remained consistent, whether compared against the untreated condition, the L3K+P3000 condition, or the scrambled control condition. Thus, it appeared that no excessive perturbations in expression were induced by the L3K+P3000 transfection reagents themselves.

### 3.2. Rlip Overexpression and Addition of Exogenous Recombinant Rlip Does Not Reverse Expression Pattern Changes Seen with Rlip Knockdown

**Figure 2:**
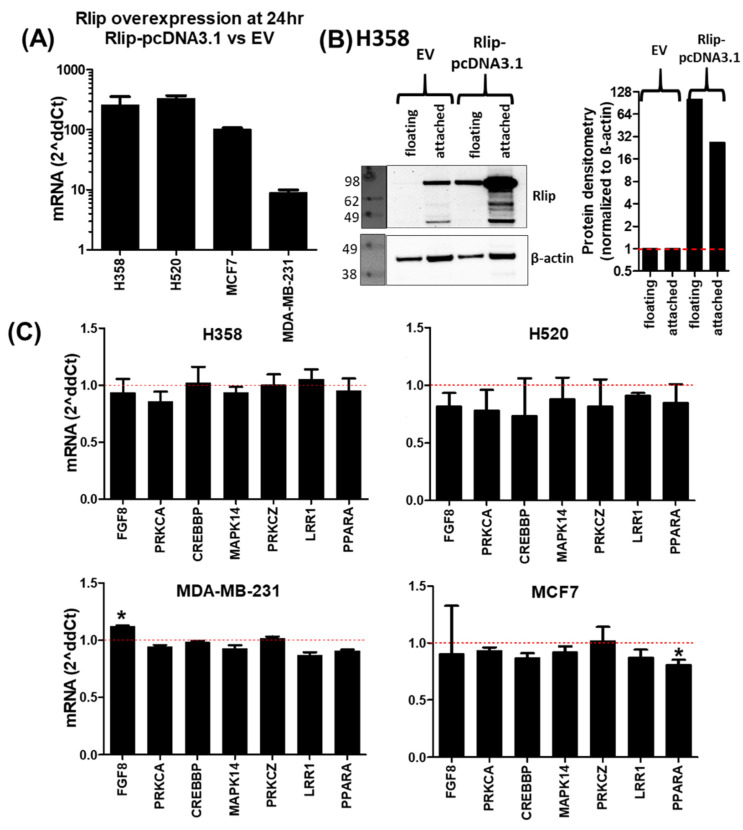
Rlip overexpression minimally alters transcription. Cells were collected 24 h following transfection with a pcDNA3.1-based Rlip overexpression plasmid or the empty vector (EV). (**A**) *RALBP1* mRNA was increased 10-fold to 300-fold with the Rlip-pcDNA3.1 plasmid relative to the EV. (**B**) Western blot in H358, confirming that increased *RALBP1* mRNA resulted in increased Rlip protein in both attached and floating cells. Densitometric analysis showed that Rlip protein was increased 27-fold in attached cells and 102-fold in floating cells at 24 h. Uncropped Western Blots and densitometry can be found at Figure S6 (**C**) Rlip overexpression resulted in relatively minor expression changes and did not result in a simple reversal of the pattern seen with Rlip knockdown by Rlip-LNA. Asterisks (*) indicate *p* < 0.05 by Student’s *t*-test when comparing mRNA expression from each Rlip-pcDNA3.1 treatment to its corresponding EV control treatment (defined as 1 and indicated by the red dotted lines). Error bars indicate SEM (*n* = 3).

**Figure 3:**
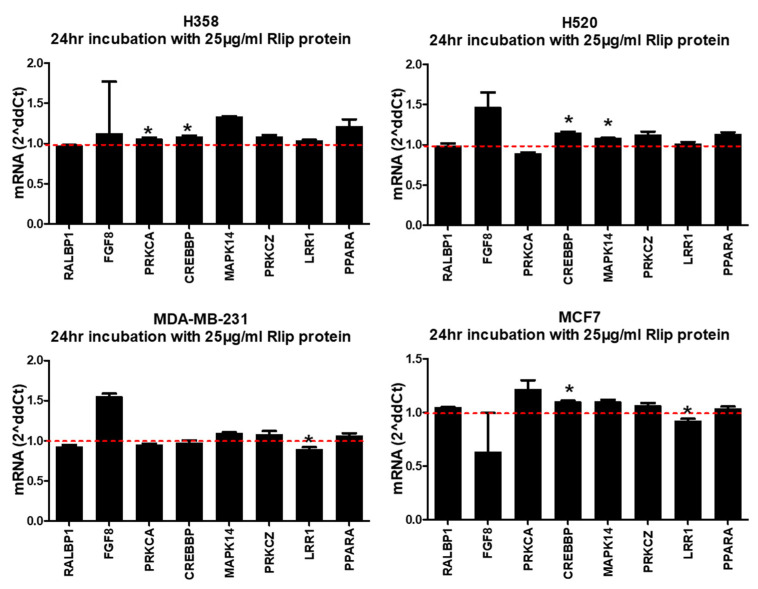
Addition of purified recombinant Rlip protein minimally alters transcription. Cells were collected 24 h following addition of 25 µg/mL (50 µg in 2 mL growth medium) purified GMP-grade recombinant Rlip protein in PBS. The results are shown as the fold-change of expression of Rlip-treated cells relative to PBS-treated controls, which were defined as 1 (indicated by red dotted lines). Asterisks (*) indicate *p* < 0.05 by Student’s *t*-test when comparing mRNA expression from each Rlip protein treatment to its corresponding PBS control treatment. Error bars indicate SEM (*n* = 3).

We have seen that Rlip knockdown is able to alter the mRNA expression of several cancer-related genes. If Rlip level influences target expression via a relatively direct mechanism, then increasing Rlip should cause opposing effects on expression. To evaluate this, we increased Rlip by (1) transfection of an Rlip expression plasmid and (2) addition of purified Rlip to the culture medium. Transfection of the Rlip-pcDNA3.1 plasmid resulted in considerable overexpression of both *RALBP1* mRNA (Figure 2A) and Rlip protein (Figure 2B). *RALBP1* mRNA was increased 10-fold (MDA-MB-231) to 300-fold (H520) with the Rlip-pcDNA3.1 plasmid relative to the empty vector (EV). We also tested whether Rlip protein was detectable following transfection of Rlip-pcDNA3.1. At 24 h post-transfection, Rlip protein was increased 25-fold in attached H358 cells and 100-fold in floating cells, and the Rlip produced by the plasmid was at the same molecular weight as the cell’s native Rlip, the band of which was detected in EV-transfected cells. In overexpressing Rlip, we expected to observe a pattern of expression changes opposite to that observed with Rlip knockdown; however, this was not observed at 24 h following the transfection of the Rlip-pcDNA3.1 plasmid (Figure 2C). To validate this result, we also treated the cells with GMP-grade recombinant Rlip protein in PBS. This is a lyophilized product produced by Terapio Corporation to mitigate or treat radiation poisoning. We inoculated 50 µg (25 µg/mL) Rlip protein directly into the cell culture medium. Again, we did not observe expression changes opposite to those induced following Rlip knockdown by Rlip-LNA (Figure 3). Treatment with recombinant Rlip protein did not alter *RALBP1* mRNA levels in any cell line. These results suggest that the transcriptional changes observed following Rlip depletion cannot be explained by a simple model whereby Rlip modulates expression in a 1:1 manner. Rather, these results support a model in which complex haploinsufficiency interactions involving Rlip result in a system where increases in Rlip do not result in effects opposite to those of decreases in Rlip. A further consideration is that a reduction in Rlip is incredibly stressful to cancer cells, as evidenced by the many publications demonstrating the cytotoxicity of Rlip knockdown, whereas the improved stress tolerance offered by Rlip overexpression would perhaps be irrelevant in the absence of stress or a further elevated metabolism. Under this scenario, once a cell has sufficient Rlip to cope with its current metabolic stresses, increasing the quantity has little impact.

### 3.3. In Vitro CpG Methylation Downregulates the Activity of Cloned Promoter CpG Islands of All Target Genes

**Figure 4:**
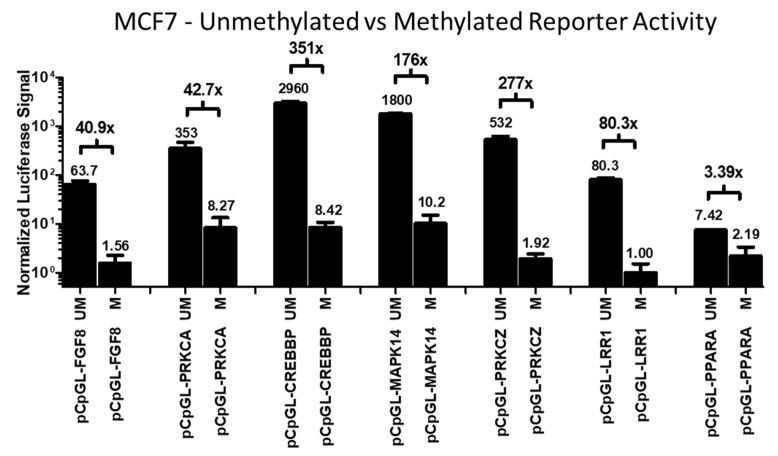
Relative activities of methylated and unmethylated pCpGL reporter constructs in MCF7 cells. Each well (96-well plate) was co-transfected with 100 ng of a methylated or unmethylated pCpGL reporter plasmid construct and 5 ng of pRL-SV40 renilla luciferase internal standard. The firefly luciferase pCpGL reporter signal was normalized to the pRL-SV40 renilla luciferase signal (firefly/renilla) in the same well. In order to emphasize the multi-log range of relative activities between all constructs, both methylated and unmethylated, all values were normalized to methylated pCpGL-LRR1, the condition with the lowest firefly/renilla signal ratio, which was defined as =1 (see values above each bar). The fold difference in signal for each methylated and unmethylated pair is shown. UM: Unmethylated. M: Methylated. Error bars indicate SEM (*n* = 2).

In order to further study the various regulatory mechanisms contributing to transcriptomic regulation by Rlip, we cloned promoter CpG island regions upstream of these same genes (*FGF8*, *PRKCA*, *CREBBP*, *MAPK14*, *PRKCZ*, *LRR1*, and *PPARA*) into the pCpGL CpG-free luciferase reporter plasmid [64]. As the pCpGL backbone plasmid lacks 5′→3′ CpG dinucleotides, these reporter constructs can only be methylated on CpG sites in the cloned promoter insert. This allowed us to study promoter-specific regulatory effects in the absence of confounding regulatory influences exerted directly by histones, miRNAs, lncRNAs, or mRNA processing. In the absence of any Rlip-altering treatments, we first evaluated the luciferase signal when the reporter constructs were unmethylated and when the reporter constructs were methylated in vitro by M.SssI methyltransferase prior to transfection. Consistent with the typical characterization of promoter methylation as a suppressive epigenetic modification, methylation greatly suppressed the luciferase signal produced by the promoters of all genes studied in this work. Figure 4 shows the fold-change in firefly luciferase signal resulting from in vitro CpG methylation of the luciferase reporter constructs before transfection into MCF7 cells. In vitro CpG methylation was similarly found to suppress the expression of reporters in H358 and H520 cells. MDA-MB-231 cells were not used for dual-luciferase assay experiments because initial pilot studies using unmethylated reporter constructs in these cells found an inadequate signal-to-noise ratio due to low luciferase signal.

### 3.4. Promoter CpG Island/Luciferase Reporters Are Differentially Responsive to Rlip Knockdown

**Figure 5:**
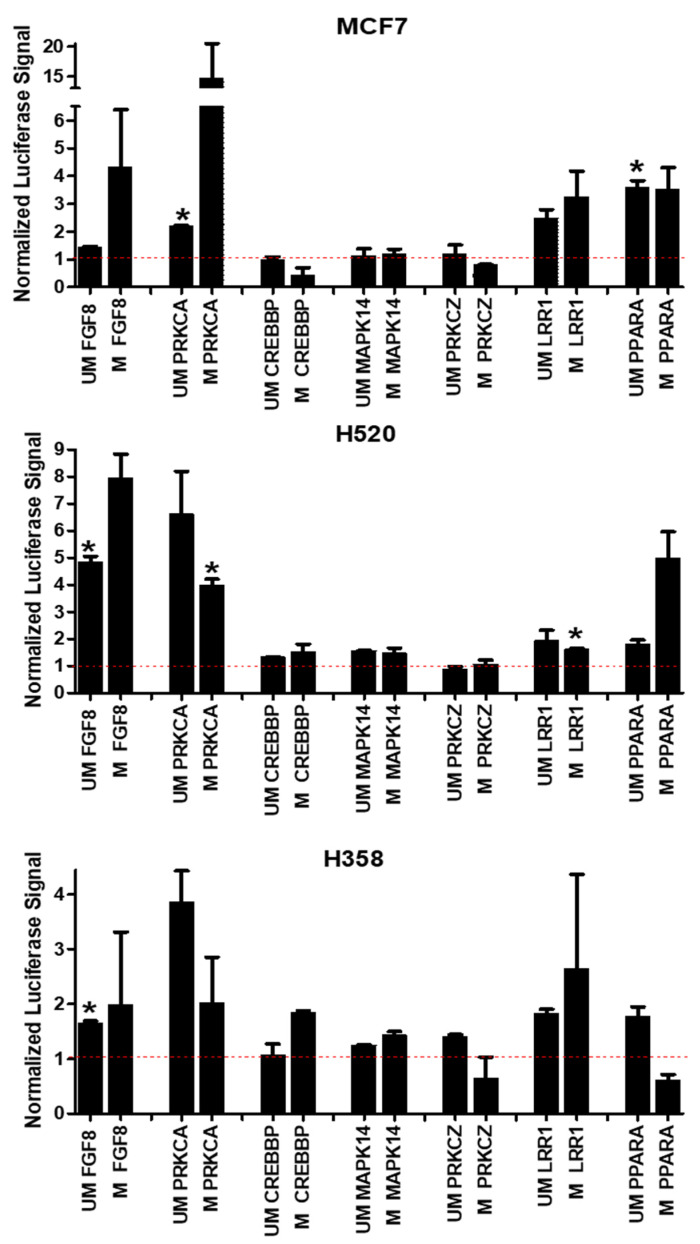
Isolated CpG islands from target genes are differentially responsive to Rlip knockdown. Promoter CpG islands upstream of the translation start site were cloned into a CpG-free firefly luciferase reporter plasmid. Methylated and unmethylated versions of the constructs were transfected into cancer cell lines using Lipofectamine 3000 with P3000. The responsiveness of promoter constructs to Rlip knockdown by an Rlip-targeted locked nucleic acid (Rlip-LNA) was evaluated relative to the scrambled controls. The bars indicate the fold-change in signal produced by a given methylated or unmethylated reporter under conditions of Rlip depletion, relative to the signal produced by the construct under the corresponding scrambled control condition, which was defined as equal to 1. To avoid overcrowded graphs, the signals produced under the scrambled condition are not shown using bars, but are instead indicated by the horizontal red dotted line at y = 1. Asterisks (*) indicate *p* < 0.05 by Student’s *t*-test when comparing luciferase signal from each Rlip-LNA treatment to its corresponding scrambled control treatment. Error bars represent SEM. UM: unmethylated. M: methylated. Error bars indicate SEM (*n* = 2).

The responsiveness of the promoter CpG island/luciferase reporter plasmids to Rlip knockdown by Rlip-LNA was evaluated with CpG cytosine residues in both the fully methylated and fully unmethylated states. The CpG island luciferase reporter constructs were transfected into MCF7, H358, and H520 cells using Lipofectamine 3000 with P3000. The FGF8, PRKCA, LRR1 and PPARA constructs were generally responsive to Rlip knockdown, while the CREBBP, MAPK14, and PRKCZ constructs were relatively unresponsive to Rlip knockdown (Figure 5). Although the degree of signal induction differed somewhat between cell lines, similar patterns of responsiveness and nonresponsiveness were generally observed across all cell lines and the patterns were also similar between the methylated and unmethylated variants of the reporter plasmids. This pattern was distinct from the pattern of responsiveness seen at the mRNA level by qRT-PCR following Rlip knockdown (Figure 1). Importantly, increased expression was observed for many fully unmethylated plasmids, which by definition cannot be upregulated by further demethylation. This is strong evidence that Rlip exerts methylation-independent regulation on gene expression. As the plasmids themselves are not subject to regulation by histone modification or chromatin remodeling and because the transcripts, which encode luciferase, are not subject to regulation by splicing, microRNAs, lncRNAs, or other transcript-specific mechanisms, the observed increases in luciferase signal following Rlip knockdown must be due to transcription factor mediated interactions which are affected by Rlip knockdown. In order to examine which transcription factors may account for the observed reporter behavior, we entered the sequence of each reporter insert into PROMO, a web-based software and database that can identify the occurrence of transcription factor binding sequences within a sequence of interest for the purpose of predicting transcription factor interactions. The results of this search are shown in Table S3. The transcription factors c-Ets-1 and C/EBPalpha were unique to the *PRKCA* and *FGF8* insert sequences, respectively. Interestingly, YY1 transcriptional repressor binding sequences were present in the highest copy number in the *PRKCZ* insert and in the lowest copy number in the *PRKCA* insert. These were the inserts with the least and greatest Rlip-responsiveness, respectively.

### 3.5. Arachidonic Acid Treatment Does Not Mimic the Target mRNA Expression Changes Seen with Rlip Knockdown

**Figure 6:**
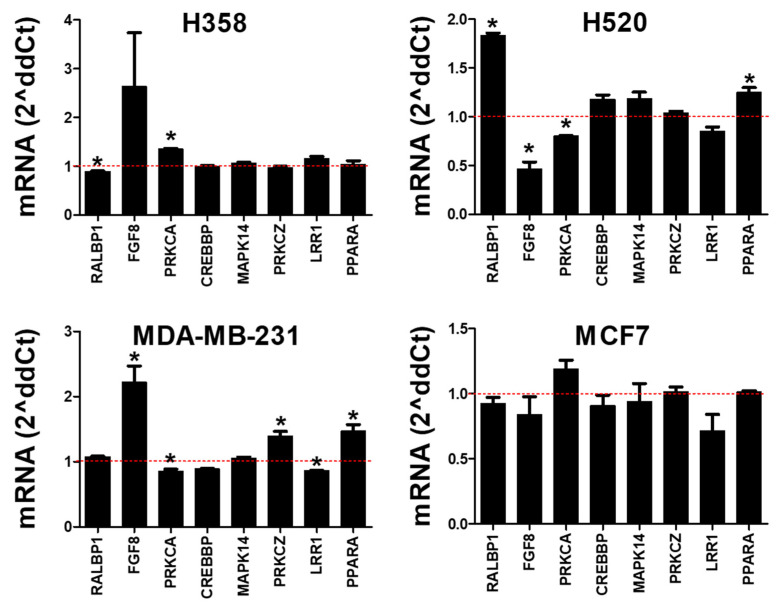
Addition of arachidonic acid minimally alters transcription. Cells were collected 24 h following addition of 150 µM arachidonic acid, a precursor of the Rlip substrate 4HNE. The results are shown as the fold-change of expression relative to DMSO-treated controls, which were defined as 1 as indicated by the red dotted lines. Asterisks (*) indicate *p* < 0.05 by Student’s *t*-test when comparing mRNA expression from each arachidonic acid treatment to its corresponding DMSO control treatment. Error bars indicate SEM (*n* = 3).

We considered the possibility that the expression changes observed following Rlip knockdown are not due to direct interactions of Rlip at all, but are perhaps due to a generally altered intracellular milieu following Rlip knockdown. It is well established that Rlip facilitates the efflux of 4HNE, an oxidative and genotoxic metabolite of omega-6 polyunsaturated fatty acids, thus we hypothesized that elevated 4HNE may recapitulate the expression effects of Rlip knockdown. We added 150 µM arachidonic acid, a precursor of 4HNE, to cell culture with the purpose of raising cellular 4HNE levels. The use of arachidonic acid as a method of raising intracellular 4HNE in cancer cells has recently been reported [67]. Aside from moderate increases in *FGF8* in H358 and MDA-MB-231, we found no notable expression changes after 24 h of incubation (Figure 6). Thus, while 4HNE accumulation following Rlip inhibition likely contributes to the cytotoxic effects of Rlip inhibition on cancer, it is unlikely to be a major driver of the transcriptomic influences of Rlip knockdown observed here. It is interesting to note that *FGF8* was induced by arachidonic acid treatment only in the two p53 non-functional cell lines. Further work is required to determine whether there is a connection between the loss of p53 and the induction of *FGF8* in response to elevated lipid peroxidation.

### 3.6. Luciferase Assay Expression Patterns Are Not Determined by Diminished Clathrin-Dependent Endocytosis (CDE) or Cell Death following Rlip Depletion

As Rlip knockdown resulted in different patterns of responsiveness by qRT-PCR and by dual luciferase assay and because cellular uptake by lipofection is reported to function via CDE, a process that involves Rlip [68], we considered whether our dual luciferase assay results may be reflective of diminished transfection efficiency owing to inhibited CDE following Rlip depletion. Transfection takes place within 4–6 h following the addition of Lipofectamine/DNA complexes to the cell culture medium. To study the possibility that Rlip knockdown blunts plasmid uptake during the transfection window, we tandem transfected the highly Rlip-LNA-responsive pCpGL-PRKCA reporter plasmid and then transfected Rlip-LNA (or scrambled control) following a 6 h delay, or vice versa, using H358 and H520 cells. Thus, in one condition, the pCpGL-PRKCA reporter was transfected first, followed 6 h later by Rlip-LNA or scrambled control, and in another condition, the Rlip-LNA or scrambled control was transfected first, followed 6 h later by the reporter plasmid. The normalized expression pattern was qualitatively similar between the two tandem transfection conditions. Transfection of Rlip-LNA or scrambled control prior to the pCpGL-PRKCA luciferase reporter resulted in a slightly larger effect size than when the reporter was transfected first. Both results led to the same interpretation regarding the Rlip responsiveness of the pCpGL-PRKCA reporter (Figure S4). We conclude that Rlip knockdown does not disrupt transfection efficiency during the assay’s transfection window.

As Rlip depletion is also known to be cytotoxic to cancer cells, we considered whether our results might be affected by differential cytotoxicity between the various combinations of pCpGL reporter constructs and Rlip-LNA (or scrambled control). To evaluate this, we performed MTT cytotoxicity assay in parallel with dual-luciferase assay following co-transfection of a *PRKCA*, *CREBBP*, or *FGF8* pCpGL reporter with Rlip-LNA (or scrambled control). We observed no notable differences in cell kill between the reporter/LNA combinations at the 24 h timepoint, despite clear differences in the Rlip-responsiveness of the luciferase reporters (Figure S5).

## 4. Discussion

The results of our previous studies demonstrated that large-scale methylomic and transcriptomic changes occur following Rlip depletion through unknown intermediate regulatory steps. In the present studies, we tested the hypothesis that specific regulatory complexes involving Rlip protein may be involved. We attempted to further characterize the downstream regulatory outcomes of Rlip depletion on several cancer-related genes with functional or pathway commonality with Rlip. Using qRT-PCR, we confirmed that Rlip knockdown does in fact alter the transcription of some of these cancer-related genes. Rlip-LNA transfection resulted in more robust *RALBP1* depletion and more robust transcriptional effects in the breast cancer cells relative to the lung cancer cells; however, this could simply be a function of the faster doubling times of the breast cancer cells, as opposed to biology specific to the cancers of origin. We found that the transcriptional regulation following Rlip depletion was not likely due to the stress of 4HNE accumulation or other effects related to arachidonic acid, except possibly in the case of *FGF8*. When upstream promoter CpG island regions of these same genes were inserted into luciferase reporter plasmids, the expression was also altered by Rlip knockdown, but in a different pattern. Using these reporter plasmids, we found that the expression was vastly altered by in vitro CpG methylation by M.SssI methyltransferase, with methylation being suppressive in all cases. However, 24 h Rlip-knockdown resulted in changes to reporter activity that were much smaller in magnitude. It is thus unlikely that cells were methylating the unmethylated reporter plasmids or demethylating the methylated reporter plasmids to any substantial degree within the 24 h assay window. Further, Rlip knockdown resulted in increased reporter signal from plasmids that were already fully unmethylated, thus activation by further demethylation was not possible. These luciferase reporter observations point toward a transcription factor-based mechanism of regulation by Rlip knockdown at 24 h, rather than a methylation-based mechanism. The distinctly different patterns between the luciferase reporter activity changes and the mRNA expression changes following Rlip knockdown also indicated that another layer of regulation, in addition to a transcription factor-mediated layer of regulation, is involved in the cell’s short-term transcriptomic response to Rlip knockdown, although it is unclear what this mechanism is.

We have previously identified Rlip-p53 interactions in neuroblastoma [15] and have also found that Rlip knockdown prevents age-acquired transcriptomic and methylomic abnormalities in p53 knockout mice [1]. These findings led to the hypothesis that Rlip knockdown may exert its transcriptomic effects by altering the ratios of regulatory complexes involving Rlip and p53. In this work, we have studied H358 and MDA-MB-231, a p53-null lung cancer cell line and a p53 mutant breast cancer cell line, respectively, for which we have found similar patterns of Rlip-responsiveness as those seen with H520 and MCF7, which are p53-functional cell lines established from lung and breast cancer, respectively. This argues that the Rlip-responsiveness demonstrated in Figure 1 and Figure 5 is p53-indepenent. Indeed, Rlip depletion suppresses malignancy just as effectively in p53-null and p53 wild-type mice, which also supports this assertion. This fact, combined with the noted Rlip-responsiveness of p53-null H358 cells and the previously mentioned observation that the upregulation of a fully unmethylated reporter plasmid can only occur via a transcription factor-based mechanism, leads to the conclusion that short-term Rlip depletion affects transcription via a methylation-independent and p53-independent transcription factor-mediated mechanism. When taken in the context of our previous findings [1,15,69], this suggests that the long-term oncopreventive regulatory effects of Rlip depletion likely operate via a mechanism independent of the effects observed here.

Cells use a variety of mechanisms to regulate expression. mRNA abundance can reflect effects based on histone remodeling, CpG methylation, transcription factors, microRNAs (miRNA), and long non-coding RNAs (lncRNA), in addition to a variety of effects involving mRNA processing (splicing, capping, and poly-A tailing), mRNA secondary structures, and modified nucleotides that can alter mRNA half-life [25,69,70,71]. On the other hand, as the reporter plasmids are non-chromosomal and produce only transcripts encoding luciferase, the reporter signal can only be subject to influence by methylation and transcription factor activity occurring at its promoter CpG island insert. By definition, target-specific posttranscriptional regulation of mRNAs, such as hairpin secondary structures, splicing, or mRNA base modifications, would not affect luciferase reporter mRNA. Neither would the luciferase mRNA transcript be subject to target-specific regulation by micro RNAs or lncRNA interactions. Further, although there have been reports of nuclear-localized reporter plasmids associating into nucleoprotein structures, it is highly unlikely that transiently transfected plasmids will recapitulate the regulatory aspects of histone modification or chromatin remodeling in a way that reflects gene regulation [72].

We have found that the expression driven by CpG island regions cloned into the pCpGL reporter vector indeed was suppressed when the plasmids were methylated, a finding that is in line with the predominant characterization of promoter methylation. We also observed that both methylated and unmethylated reporters were typically equivalently affected by Rlip knockdown. Rlip knockdown tended to upregulate the expression of both methylated and unmethylated luciferase reporters driven by *PRKCA*-, *FGF8*-, *LRR1*-, and *PPARA*-derived CpG islands, while the *CREBBP*-, *PRKCZ*-, and *MAPK14*-derived CpG islands resulted in relatively little change in luciferase signal following Rlip knockdown. The reporter plasmids used for experiments were either completely unmethylated (as produced by the PIR1 *E. coli* strain used in cloning) or completely methylated (as expected following M.SssI methyltransferase treatment) at the time of transfection. This was verified by digestion with the methylation-sensitive restriction enzyme HpaII, which showed complete protection or complete digestion dependent on methylation status. It is unclear to what extent the DNA methyltransferase (DNMT) or ten-eleven translocation (TET) proteins can methylate or demethylate, respectively, reporter plasmids during the assay timeframe; however, given our assay design, it is neither possible for cells to further de-methylate the unmethylated constructs nor to further methylate the methylated constructs. Therefore, as Rlip inhibition tended to similarly affect the unmethylated and methylated constructs, it is unlikely that CpG methylation or demethylation was driving the observed changes in luciferase reporter output following Rlip knockdown.

A possible limitation in the interpretation of our results, in the context of previous findings on congenitally Rlip-deficient mice, lies in the very short 24 h assay duration. It is possible that evidence of methylomic regulatory changes would have arisen with longer assay durations; however, prior studies on the polyphenolic phytoalexin resveratrol in MDA-MB-231 breast cancer cells indicate that both methylation and demethylation changes occurred within 24 h of dosing, with minimal further changes observed at 48 h [73]. Additionally, it has been shown that the DNMT inhibitors 5-azacytidine and RG108 are able to elicit expression changes in Hep3B hepatocellular carcinoma cells within 24 h of dosing [74], thus epigenetic alterations to cytosine methylation can clearly arise quickly. In our prior work, weekly treatments to deplete Rlip were initiated on 8-week-old mice and continued for the duration of their lifespans, up to 48 weeks [1], making for a considerably longer assay duration. Unfortunately, long-term in vitro exposure of cancer cell lines to Rlip knockdown results in high levels of cell kill, which would be problematic for the assays used here.

Degree of knockdown is another limitation, as greater knockdown may have elicited different effects. In three of four cell lines, we attained only ~50% Rlip knockdown at 24 h; however, it is well established that ~50% depletion of Rlip by heterozygous Rlip knockout shows good anticancer activity and is even optimal, as evidenced by the phenotype and survival of heterozygous Rlip knockout mice [1,31]. The level of knockdown attained in our experiments should be a good approximation of the Rlip levels in such heterozygous knockout mice [31]. A final limitation of the assay is that only promoter CpG island regions upstream of the translation start site were cloned into the pCpGL luciferase reporter plasmid. This design was chosen for the purpose of avoiding complications from fusion proteins consisting of luciferase and large fragments translated from exon 1 and intron 1 of the target gene.

Surprisingly, the expression responses as assessed by qRT-PCR were highly consistent across the four cell lines following Rlip knockdown. Similarly, the expression responses as assessed by the luciferase reporters were also highly consistent across the three cell lines tested. This argues that the observed effects may be inherent to the gene/promoter structures and are little affected by tissue of origin or by cell-line-specific differences in p53 deletion, estrogen receptor expression, or *KRAS* mutation. This consistency across cell lines is in line with the broad-spectrum anticancer activity previously reported for Rlip inhibition methods, which have demonstrated efficacy against preclinical models of melanoma; neuroblastoma; glioblastoma; and cancers of the breast, lung, colon, kidney, and prostate [12,13,15,16,17,18,19].

Of the gene targets studied in this work, perhaps the most interesting pattern was seen with *FGF8*. Although Rlip knockdown moderately downregulated the other targets at 24 h following transfection, FGF8 expression was massively increased. This was unexpected given the oncogenic properties typically attributed to FGF8 and the cancer suppressive activities seen with Rlip knockdown. However, the FGF/FGFR family of signaling structures is quite complex, with the Ras/MAPK pathway being a prominent signal effector [75,76]; There are approximately 30 secreted FGF proteins and approximately 49 FGFR receptors, if the distinct isoforms encoded by the 22 FGF genes and 5 FGFR genes are counted [77,78]. Each FGF receptor has a distinct profile of affinities for the various secreted FGFs [79]. Given this complexity, FGF/FGFR signaling interactions operate in both oncogenic and tumor suppressive contexts [79,80]. It is possible that the observed *FGF8* upregulation in our experiments is due to a compensatory upregulation resulting from interference with FGFR signaling. While the gene targets studied in this work are typically considered to have oncogenic activities, many of them have also been reported to mediate tumor suppressor functions [40,80]. In some cases, tissue-specific or cancer-specific contexts can determine the oncogenic versus tumor suppressive effects of a gene, and in other cases, mutations can convert a protein with tumor suppressive function into one with an oncogenic function, similar to the haploinsufficiency interactions that result in broad and opposite effects on apoptosis by alterations in the level of a single BH domain protein. Thus, we must bear in mind that expression levels alone can be insufficient to draw conclusions regarding functional outcome when attempting to interpret the significance of specific changes in expression presented in this paper.

## 5. Conclusions

The key conclusions of this work are that Rlip inhibition clearly results in changes in the expression of the majority of these cancer-related genes in all cell lines tested, and it appears that, at the 24 h timepoint, at least two distinct mechanisms are operative. One mechanism is dependent on transcription factors, and a second mechanism is clearly operative, but cannot be explained by methylation or transcription factors. To further clarify this, future studies should prioritize the deletion of candidate Rlip-responsive transcription factor binding sites from cloned promoter inserts and the examination of how Rlip depletion affects histone acetylation and microRNA expression. Further, Rlip-responsiveness following 24 h knockdown appears to be independent of 4HNE accumulation and p53. Our findings support Rlip as a broadly important protein in determining the expression of cancer-related genes. This work, in combination with previously reported findings, supports the operation of a haploinsufficiency mechanism and the presence of at least two distinct modes of action, which elicit the oncopreventive effects of long-term Rlip depletion and the short-term chemotherapeutic anticancer effects of Rlip depletion.

## Data Availability

The data presented in this study are available in the article and supplementary material.

## Supplementary Materials

The following supporting information can be downloaded at: https://www.mdpi.com/article/10.3390/cancers14030527/s1, Table S1: qRT-PCR primer sequences. Table S2: Primer sequences for promoter CpG amplification. Table S3: Predicted transcription factor binding sites occurring in the insert sequences of the pCpGL luciferase reporter constructs. Figure S1: Cloned insert quality control. Figure S2: Verification of methylation by M.SssI methyltransferase. Figure S3: Vehicle effects on mRNA expression in MCF7 cells. Figure S4: Impact of Rlip-LNA on reporter transfection efficiency. Figure S5: Luciferase reporter expression patterns are not driven by cytotoxicity. Figure S6: Densitometric quantitation of Rlip overexpression by the Rlip-pcDNA3.1 expression plasmid in H358 cells.
