# Supplementary material for: Rlip Depletion Alters Oncogene Transcription at Multiple Distinct Regulatory Levels
Source: Cancers (Basel). 2022 Jan 21;14(3):527. doi: 10.3390/cancers14030527 (PMC8833773; doi:10.3390/cancers14030527)
Supplement: Supplementary file 1 [file cancers-14-00527-s001.zip › cancers-1511542 supplementary proofread/cancers-1511542 supplementary proofread.pdf]

Table S1. qRT-PCR primer sequences.

Primer:	Sequence
LRR1 Forward[a]	5′-GGGACCCGCTATGAGCTAAG-3′
LRR1 Reverse[a]	5′-CCTTTAACCGAACAGTGGCTTT-3′
PPARA Forward[a]	5′-ATGGTGGACACGGAAAGCC-3′
PPARA Reverse[a]	5′-CGATGGATTGCGAAATCTCTTGG-3′
PRKCA Forward[a]	5′-TGGACTTATCCATCAAGGGATGA-3′
PRKCA Reverse[a]	5′-AGTGTGATCCATTCCGCAGAG-3′
PRKCZ Forward[a]	5′-CTTACATTTCCTCATCCCGGAAG-3′
PRKCZ Reverse[a]	5′-TTCACCACTTTCATGGCGTAAA-3′
MAPK14 Forward[a]	5′-TCAGTCCATCATTCATGCGAAA-3′
MAPK14 Reverse[a]	5′-AACGTCCAACAGACCAATCAC-3′
CREBBP Forward[a]	5′-CGGCTCTAGTATCAACCCAGG-3′
CREBBP Reverse[a]	5′-TTTTGTGCTTGCGGATTCAGT-3′
FGF8 Forward[a]	5′-GACCCCTTCGCAAAGCTCAT-3′
FGF8 Reverse[a]	5′-CCGTTGCTCTTGGCGATCA-3′
RALBP1 Forward[a]	5′-TGAAGTGTGAAGGCATCTACAGA-3′
RALBP1 Reverse[a]	5′-TGGCTACAGTGTTAGGCTCATAG-3′
GAPDH Forward[b]	5′-TGGAAGGACTCATGACCACAG-3′
GAPDH Reverse[b]	5′-CAGCTCAGGGATGACCTTGC-3′

[a]Primer sequences from Harvard PrimerBank [b]Primer designed using NCBI Primer-BLAST

Table S2. Primer sequences for promoter CpG amplification.

Primer:	Sequence	Restriction Site	Amplicon Length[a]
LRR1 Forward	5′-ACACAGATCTGAGAGCAGCCACCAAAACCA-3′	BglII	512
LRR1 Reverse	5′-ATGACCATGGACGTCAAGCTGGCTTTGAAAC-3′	NcoI	
PPARA Forward	5′-TACTGGATCCCGTCACGGCCCGAACAAAG-3′	BamHI	837
PPARA Reverse	5′-ATGAAAGCTTAGCCAGTGTCCCGAGGC-3′	HindIII	
PRKCA Forward	5′-CTCAGGATCCGGTCCTGAGGATGGGGAAGG-3′	BamHI	886
PRKCA Reverse	5′-CTAGAAGCTTCAACCACCTCTTGCCTCCG-3′	HindIII	
PRKCZ Forward	5′-CAGACTGCAGATGCCGCGTCCTTGACCT-3′	PstI	786
PRKCZ Reverse	5′-GATAAAGCTTGTCAGCGCTGCGCCC-3′	HindIII	
MAPK14 Forward	5′-CCTGCTGCAGTGGAATTTGGGGGTTCAGGTG-3′	PstI	746

MAPK14 Reverse	5′-CTAGAAGCTTCAAGAAGGTGGCCCTGTGG-3′	HindIII	
CREBBP Forward	5′-TGATAGATCTCCTGGTCGCCATCAGAACAA-3′	BglII	1081
CREBBP Reverse	5′-AGTCCCATGGTCACCTGCTCGCGAAAACA-3′	NcoI	
FGF8 Forward	5′-ATAAAGATCTAGCCATTCGCTGCATGGTTA-3′	BglII	757
FGF8 Reverse	5′-ACACCCATGGCCCGGGGCACCGAGAG-3′	NcoI	

[a]Number of genomic base pairs amplified (value excludes base pairs of restriction site adaptors).

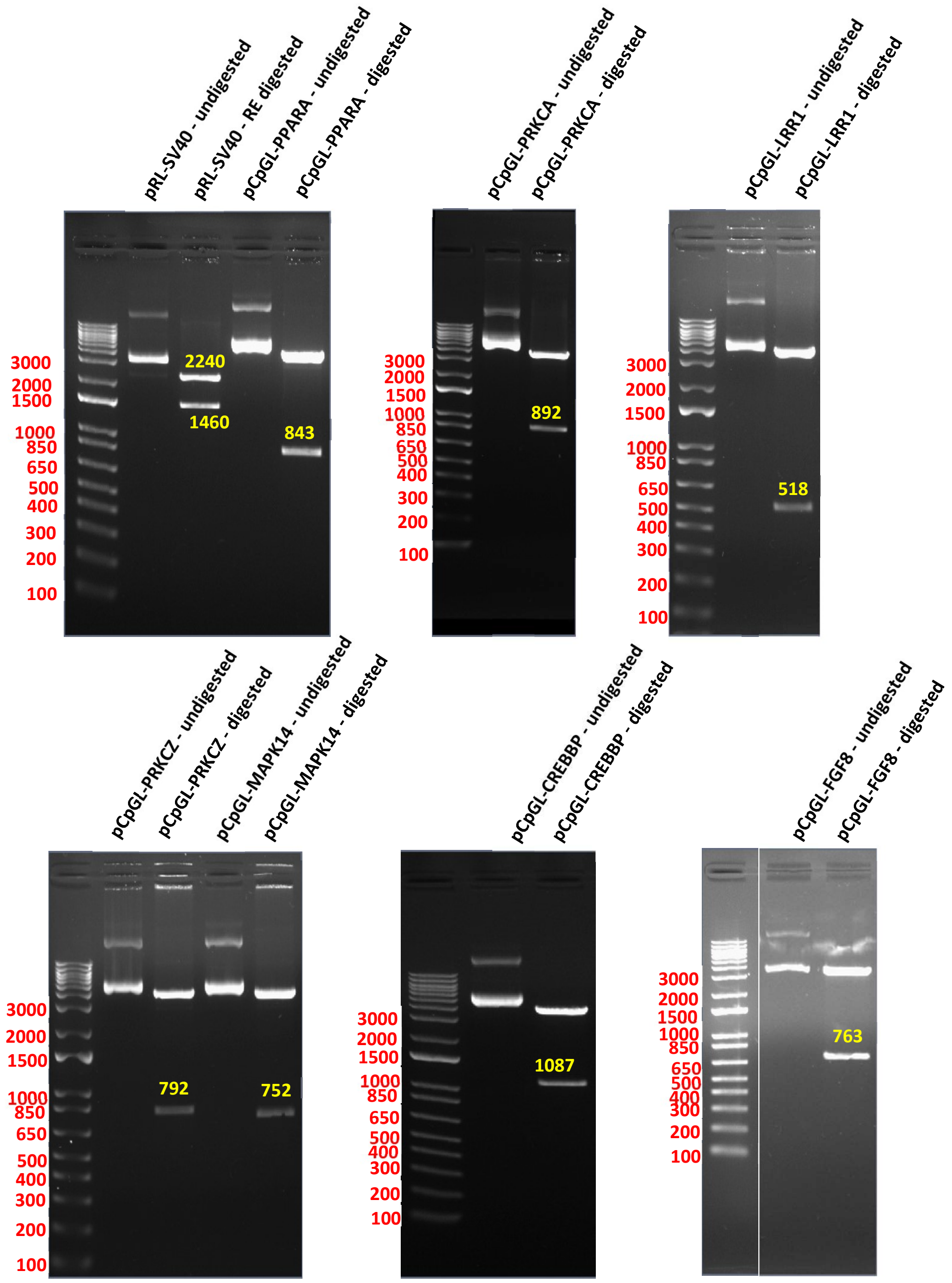

Figure S1. Cloned insert quality control. After purification of each pCpGL reporter plasmid construct from E. coli, restriction enzymes were used to remove the cloned insert for gel analysis. Digestion with restriction enzymes yielded a single band of the expected size (indicated in yellow), along with the higher MW band of the pCpGL vector. Additionally, to confirm its identity before use, Promega's pRL-SV40 Renilla luciferase loading control plasmid was restriction digested (HindIII and BamHI), resulting in the expected fragment sizes (top left panel). Invitrogen's TrackIt 1KB plus DNA ladder is shown in red.

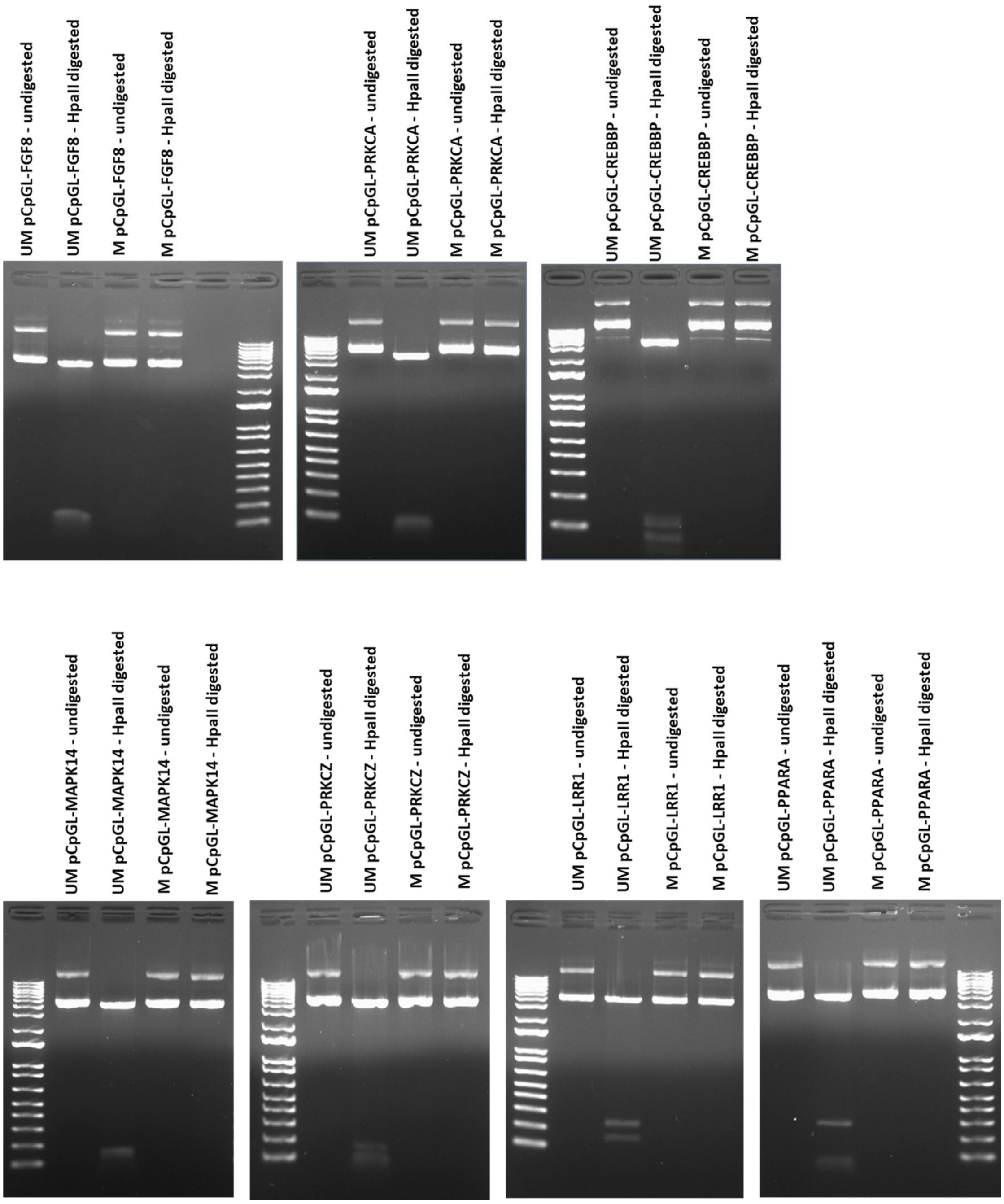

Figure S2. Verification of methylation by M.SssI methyltransferase. Plasmids methylated in vitro show complete protection from digestion by HpaII, a methylation-sensitive restriction enzyme. The patterns of 2 or 3 high molecular weight bands are characteristic of circular plasmids which can be supercoiled or relaxed (nicked), causing variable rates of migration and multiple distinct bands. When a plasmid is linearized by restriction digestion the fragments migrate uniformly as single bands, as is seen only with HpaII digestion of the unmethylated plasmids. UM: Unmethylated. M: Methylated.

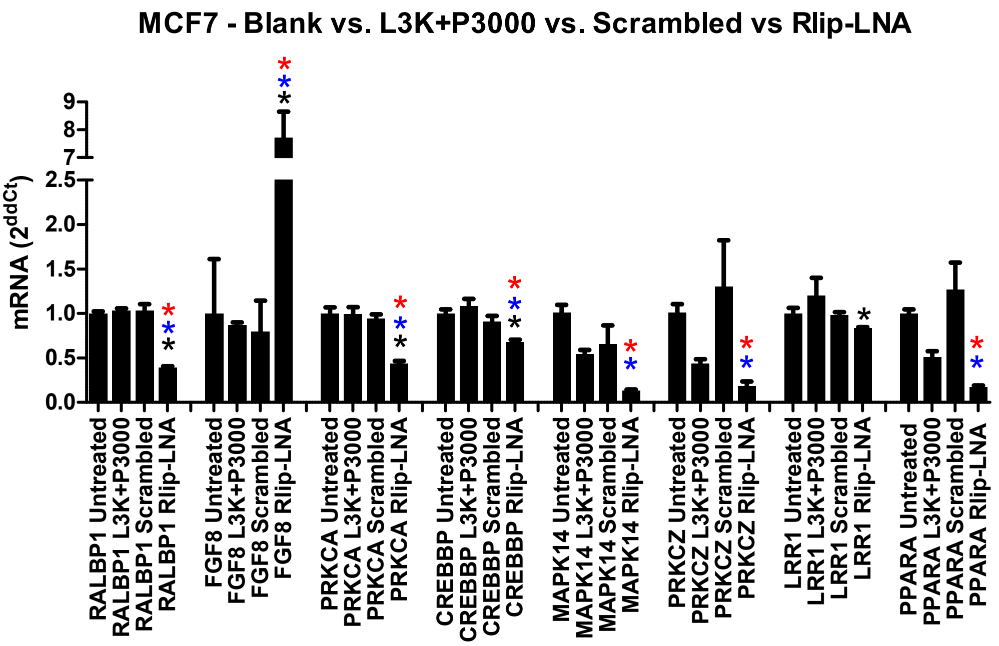

Figure S3. Vehicle effects on mRNA expression in MCF7 cells. mRNA expression in MCF7 cells exposed only to the Lipofectamine 3000 and P3000 (L3K+P3000) transfection reagents, cells transfected with scrambled control, and cells transfected with Rlip-LNA were 2^{ddCt} normalized to untreated cells (defined as 1). For *FGF8*, *PRKCA*, *CREBBP*, and *LRR1* no change in mRNA expression was observed following exposure to L3K+P3000. Moderate deceases in mRNA were observed for *MAPK14*, *PRKCZ*, and *PPARA* in response to L3K+P3000, however the direction of all regulatory effects on expression due to Rlip-LNA were consistent, whether normalized to the untreated condition, the L3K+P3000 condition, or the scrambled condition. Red asterisks (*) indicate $p < 0.05$ by Student's t-test when Rlip-LNA treatment is compared to the untreated cells. Blue asterisks (*) indicate $p < 0.05$ by Student's t-test when Rlip-LNA treatment is compared to cells treated with L3K+P3000. Black asterisks (*) indicate $p < 0.05$ by Student's t-test when Rlip-LNA treatment is compared to cells transfected with scrambled control LNA, respectively. Error bars indicate SEM (n = 3).

Table S3. Predicted transcription factor binding sites occurring in the insert sequences of the pCpGL luciferase reporter constructs. Average fold change in luciferase signal following Rlip depletion is shown at the top and incorporates the activities of both the methylated and unmethylated variants of each reporter construct in MCF7, H358, and H520 cells. PROMO was set to identify sequences which exactly match those previously reported to be recognized by the transcription factors.

	PRKCZ	CREBBP	MAPK14	LRR1	PPARA	FGF8	PRKCA
**Avgerage fold induction**	1.00	1.21	1.35	2.29	2.73	3.70	5.56
				**Number of occurrences**			
-	PRKCZ	CREBBP	MAPK14	LRR1	PPARA	FGF8	PRKCA
**ER-alpha**	3	-	-	1	1	2	-
**IRF-2**	1	-	-	-	-	-	-
**C/EBPbeta**	3	5	8	4	10	1	5
**GR-alpha**	3	-	1	2	1	2	-
**YY1**	5	3	2	1	3	3	1
**ENKTF-1**	1	-	-	-	-	-	1

**NF-1**	2	-	-	-	1	-	-
**ETF**	1	-	-	-	-	1	-
**WT1**	1	-	-	1	-	2	2
**GCF**	3	4	-	1	3	2	4
**Pax-5**	2	11	3	2	5	5	11
**AP-2alphaA**	-	2	1	1	-	-	-
**TFII-I**	-	2	1	1	-	2	1
**Sp1**	-	2	-	-	1	1	-
**FOXP3**	-	1	-	1	1	-	-
**p53**	-	1	1	-	1	-	2
**TFIID**	-	2	-	-	-	-	-
**NFI/CTF**	-	1	-	-	-	-	-
**GR-beta**	-	-	1	-	-	-	-
**RXR-alpha**	-	-	1	-	-	-	-
**E2F-1**	-	-	-	1	-	-	-
**STAT4**	-	-	-	1	-	-	-
**C/EBPalpha**	-	-	-	-	-	1	-
**c-Ets-1**	-	-	-	-	-	-	1

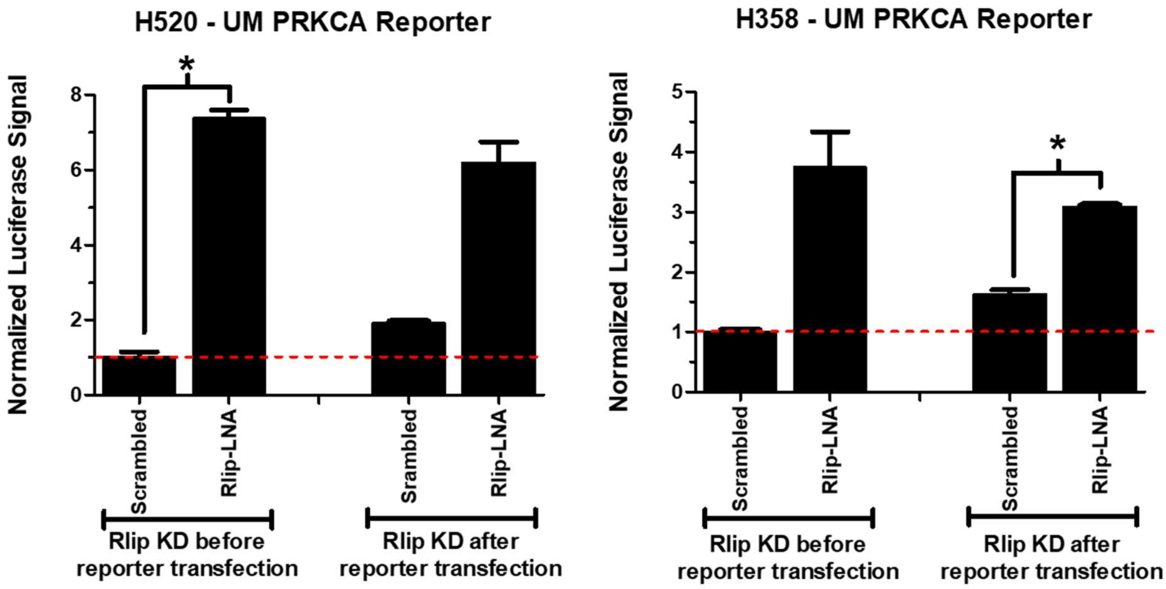

Figure S4. Impact of Rlip-LNA on reporter transfection efficiency. We considered that inhibition of clathrin-dependent endocytosis (CDE) by Rlip depletion may confound results by interfering with transfection during the 4-6 hour lipofection transfection window. Using H520 and H358 lung cancer cells, we performed a tandem transfection in which the unmethylated pCpGL-PRKCA reporter was transfected 6 hours prior to Rlip-LNA (or scramble), thus Rlip-LNA could not interfere in the transfection of the reporter. In parallel we performed the opposite tandem transfection, with Rlip-LNA (or scramble) transfection preceding pCpGL-PRKCA transfection by 6 hours. The responsiveness of the pCpGL-PRKCA reporter to knockdown by Rlip-LNA was similar under both conditions. Note the distinct linear axis scales. Asterisks (*) indicate p < 0.05 by Student's t-test. Error bars indicate SEM (n = 2). UM: Unmethylated. M: Methylated.

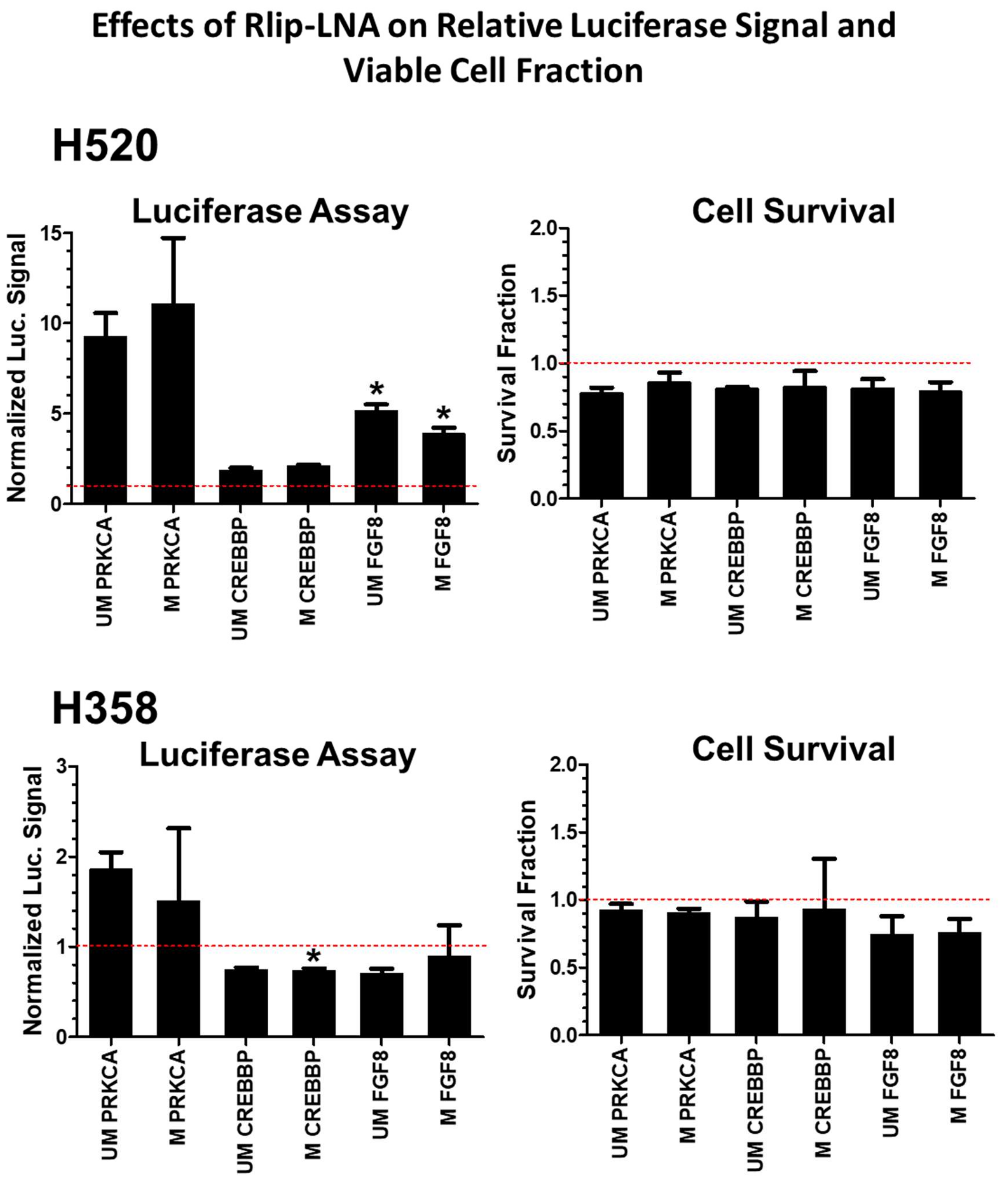

Figure S5. Luciferase reporter expression patterns are not driven by cytotoxicity. We evaluated whether the cytotoxicity or differences in cell numbers due to Rlip-LNA or reporter plasmid transfections could be affecting the observed expression results. The effect of Rlip knockdown on luciferase reporter signal for the *PRKCA*, *CREBBP*, and *FGF8* constructs was measured in H520 and H358 lung cancer cells. In parallel, the cytotoxicity to the cells was measured by MTT assay. The reporter responsiveness (left panels) and cytotoxicity (right panels) following co-transfection of Rlip-LNA and reporters are shown relative to that observed for the corresponding co-transfected scrambled control and reporters, which were defined as 1 and are indicated by the red dotted lines. The effects of Rlip knockdown on cytotoxicity at 24 hours were minimal and did not vary by the identity or methylation state of the co-transfected reporter plasmid. Thus, the luciferase expression patterns do not appear to be a reflection of differential cell death or growth, and luciferase protein itself was not cytotoxic to the cells at the levels produced by the reporter

plasmids used in the study. Asterisks (*) indicate $p < 0.05$ by Student's t-test when comparing luciferase signal or survival fraction from each Rlip-LNA treatment to its corresponding scrambled control treatment. Error bars indicate SEM (n = 2). UM: Unmethylated. M: Methylated.

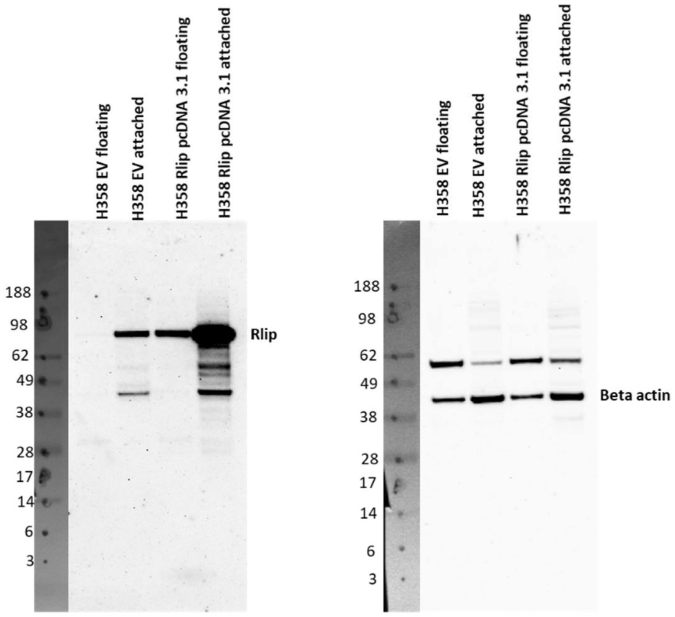

	H358 EV floating	H358 EV attached	H358 Rlip pcDNA3.1 floating	H358 Rlip pcDNA3.1 attached
Rlip densitometry	89	4185	6546	112282
B actin densitometry	8964	25537	6452	25707
Rlip/B actin	0.009929	0.16388	1.014569	4.36776
Fold change Rlip-pcDNA3.1 vs EV			102.1865	26.65221

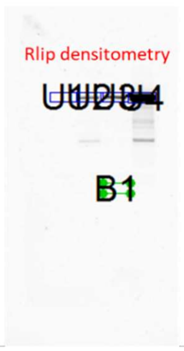

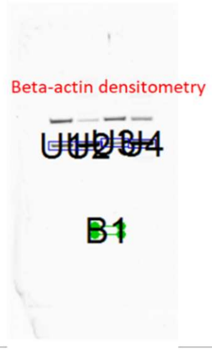

Index	Name	Volume INT*mm2	Adj. Vol. INT*mm2	% Adj. Vol.	Concentration	Area mm2
1	U1	2280.004883	89.11743164	0.07	N/A	3.6621094
2	U2	6376.342773	4185.455322	3.40	N/A	3.6621094
3	U3	8736.590576	6545.703125	5.32	N/A	3.6621094
4	U4	114472.8271	112281.9397	91.21	N/A	3.6621094
5	B1	2190.887451	0.000000000	N/A	N/A	3.6621094

Index	Name	Volume INT*mm2	Adj. Vol. INT*mm2	% Adj. Vol.	Concentration	Area mm2
1	U1	10637.82349	8963.684082	13.45	N/A	2.9296875
2	U2	27210.67505	25536.53564	38.31	N/A	2.9296875
3	U3	8126.184082	6452.044678	9.68	N/A	2.9296875
4	U4	27380.88989	25706.75049	38.56	N/A	2.9296875
5	B1	1674.139404	0.000000000	N/A	N/A	2.9296875

Figure S6: Densitometric quantitation of Rlip overexpression by the Rlip-pcDNA3.1 expression plasmid in H358 cells. Rlip and Beta actin bands were quantified using Bio-Rad's Quantity One 1-D Analysis Software. Background adjusted volumes (red boxes on lower panels) for Rlip and beta-actin were used to calculate the fold-change observed in attached and floating cells resulting from transfection of the Rlip-pcDNA3.1 plasmid, relative to the empty vector (EV).
